# Effects of Grazing Management and Cattle on Aquatic Habitat Use by the Anuran *Pseudopaludicola mystacalis* in Agro-Savannah Landscapes

**DOI:** 10.1371/journal.pone.0163094

**Published:** 2016-09-22

**Authors:** Rodolfo M. Pelinson, Michel V. Garey, Denise C. Rossa-Feres

**Affiliations:** 1 Programa de Pós-Graduação em Biologia Animal, Instituto de Biociências, Letras e Ciências Exatas, Universidade Estadual Paulista “Júlio de Mesquita Filho” - UNESP, São José do Rio Preto, São Paulo, Brazil; 2 Instituto Latino-Americano de Ciências da Vida e da Natureza, Universidade Federal da Integração Latino-Americana - UNILA, Foz do Iguaçu, Paraná, Brazil; 3 Departamento de Zoologia e Botânica, Instituto de Biociências, Letras e Ciências Exatas, Universidade Estadual Paulista “Júlio de Mesquita Filho” - UNESP, São José do Rio Preto, São Paulo, Brazil; Universitat Zurich, SWITZERLAND

## Abstract

Because of their strong dependence on the environment, the spatial distribution of pond-breeding amphibians can be greatly influenced by anthropogenic habitat alteration. In some agricultural landscapes in Brazil, the anuran *Pseudopaludicola mystacalis* appears to be highly influenced by land use. Because adult males and tadpoles of this species are usually found in marshy areas with cattle hoof prints, we hypothesized that *P*. *mystacalis* preferentially occupies aquatic habitats with marshy areas that are trampled by cattle. To test our hypothesis, we assessed whether the occurrence of *P*. *mystacalis* is associated with the presence of cattle and trampled marshy areas, and which environmental features best explain the spatial distribution and abundance of *P*. *mystacalis*. To do so, we sampled 38 aquatic habitats in an area intensely used for livestock in southeastern Brazil. We found that the presence of cattle and trampled marshy areas in aquatic habitats are positively associated to *P*. *mystacalis* occurrence. Additionally, the abundance of calling males is better predicted by variables of landscape and local habitat structure. Specifically, the size of trampled marshy areas and the proportion of herbaceous vegetation within the aquatic habitat are positively associated with abundance, while distance to nearest aquatic habitat are negatively associated with abundance of calling males. All three of these variables can be directly or indirectly linked to the presence of cattle or grazing management. Therefore, this work shows evidence that *Pseudopaludicola mystacalis* is positively influenced by grazing management with cattle, and draws attention to other unknown potential consequences of different land use to fresh water diversity.

## Introduction

Amphibians have recently experienced population declines due to habitat modification, emergent diseases, climate change, aquatic pollution, widespread introduction of predatory fish, acidification of aquatic habitats, and all their possible interactions [1,2]. More specifically, habitat loss and modification are the most important causes of amphibian declines [1]. Because of their strong dependence on the environment, the spatial distribution of pond-breeding amphibians can be expected to follow the habitat selection hypotheses (*e*.*g*. [3–5]), which assumes that individuals tend to select habitats that provide them with higher fitness [6–9]. This selection process can depend on abiotic features (*e*.*g*. environmental features), biotic interactions (*e*.*g*. such as the presence of predators and/or competitors) [7,10] and dispersal ability [9]. In the case of pond-breeding amphibians, structural complexity and hydroperiod of the aquatic habitats are crucially important [4,5,11,12]. For example, the hydroperiod of ponds determines the degree of exposure to drying or to certain predators [12,13], while structural complexity determines the availability of breeding sites and shelter from predators [14–18]. Therefore, alterations in the structural complexity of aquatic habitats can severely affect spatial distribution of amphibians (*e*.*g*. [4,11,19]).

Within this context, cattle grazing has recently received much attention because of its ambiguous impacts on pond-breeding amphibians and the overall diversity of freshwater habitats (*e*.*g*. [20–22]). Landscapes dominated by pasture can be harsh environments for amphibians. The absence of sheltering and shading structures (*e*.*g*. trees and shrubs) can increase air and soil temperature and decrease humidity, directly and negatively affecting amphibian performance and, therefore, constraining their dispersal ability even for anurans associated with open landscapes [23]. Accordingly, some recent studies have shown that some landscape features, such as distance to the nearest forest patch or to other aquatic habitats, can influence abundance and spatial distribution of many amphibian species [4,23]. However, farmland ponds are still intensively used as reproductive sites by amphibians, mostly because these ponds are often the only aquatic habitats available in agricultural landscapes [4,24]. In these ponds, cattle can negatively impact amphibians by altering water quality and reducing the number of breeding and foraging sites [20]. For example, the green frog (*Lithobathes clamitans*, referred to as *Rana clamitans* in Burton et al. [20]) was found to have fewer post metamorphic individuals in ponds where cattle were present than in those where cattle were absent, probably due to a reduction in vegetation height and cover [20]. In the other hand, the opposite pattern was found for the American toad (*Anaxyrus americanus*, referred to as *Bufo americanus* in Burton et al. [20]). Therefore, although pastures are a harsh environment to amphibians, cattle grazing can also positively impact some species that are resistant to these environmental changes [20,25].

Here we propose the hypothesis that the presence of cattle can positively influence the pond breeding anuran *Pseudopaludicola mystacalis* (Leptodactylidae). *Pseudopaludicola mystacalis* is usually found along marshy areas of ponds or in marshes with herbaceous vegetation. Tadpoles of this species complete their development in small, individual puddles (10 to 13 cm diameter) located in these marshy areas (referred to as *Pseudopaludicola* aff. *falcipes* in Sousa et al [26]). The interest thing, however, is that in northwestern São Paulo State, Brazil, these small puddles are formed, in most cases, by cattle trampling [26]. Therefore, we propose that *P*. *mystacalis* selects aquatic habitats with shallow marshy areas that are created by cattle trampling. Specifically, we predict that: (1) the occurrence of *Pseudopaludicola mystacalis* is influenced both by the access of cattle and the presence of trampled marshy areas in aquatic habitats; and (2) attributes of the physical structure of aquatic habitats, such as the size of trampled marshy areas, are more important than attributes of the landscape or water quality in explaining the abundance of calling males of *P*. *mystacalis*.

## Material and Methods

### Ethics statement

Because our sampling method did not involve any kind of animal manipulation (see study design section) approval from an ethics committee was not required. This study did not involve any endangered or protected species and all sampled aquatic habitats were located on private land, so each sampling was done with landowner permission.

### Study area

**Fig 1:**
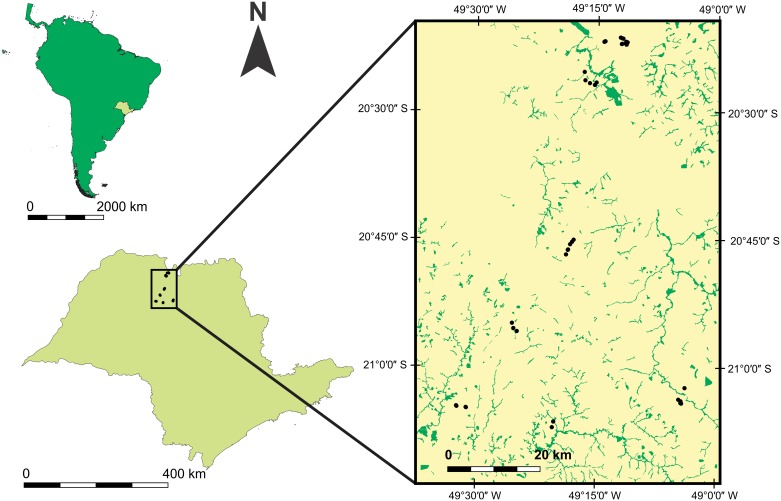
Distribution of studied aquatic habitats. Black dots represent the studied aquatic habitats in northwestern São Paulo State in southeastern Brazil. Green areas in the inset represent remaining forest whereas yellow areas represent open areas.

The study was performed in northwestern São Paulo State in southeastern Brazil (Fig 1). The region was originally dominated by mesophytic semideciduous forest (Atlantic Forest) and patches of Brazilian savanna (Cerrado) [27]. However, this vegetation has been severely reduced by agricultural and livestock activities and only small forest fragments, corresponding to only 5% of the original forest cover, remain [28]. According to the last agricultural census, the state of São Paulo contains about 7 million hectares of pasture land composed of about 41% natural pasture and 59% planted grasses [29,30]. These pasture lands host almost 11 million cattle, horses and buffaloes, for a density of about 1.6 cattle per hectare [29]. The region’s climate is hot and humid, and characterized by well-defined wet and dry seasons (Köppen’s Aw [31]). Total annual rainfall varies from about 1100 to 1300 mm and monthly average temperature varies from 20°C in the coldest month to 27°C in the hottest month [32]. The wet season occurs between October and March [33], in which about 85% of the total annual rainfall is concentrated [32].

### Sampling design

We assessed the abundance of calling males in 38 aquatic habitats (ponds and marshes) during one rainy season (September 2012 to March 2013). Distance between aquatic habitats varied from 20 m to 78 km (Fig 1), but most of the sampled habitats were more than 80 m apart with exception of two pairs of habitats that were 50 and 20 m apart (see more in S1 Table). We sampled each aquatic habitat three times during the rainy season (S2 Table): once at the beginning (September 2012 to October 2012); once in the middle (November 2012 to January 2013) and once at the end (February 2013 to March 2013). Environmental variables and geographic coordinates of each aquatic habitat were recorded one time in the middle of the rainy season before dark (S2 Table). Sampling was performed using call surveys at breeding habitats [34] between 04:00 p.m. and 11:00 p.m. (S2 Table) because this is the time period that males of *P*. *mystacalis* are usually found calling [35]. One researcher (RMP) walked slowly along the entire perimeter of each aquatic habitat and recorded the number of calling males. To avoid sampling bias because of the variable period of vocalization of *P*. *mystacalis*, each aquatic habitat was sampled at least one time during daylight (before 7:00 p.m.) and at least one time after dark (after 7:00 p.m.).

### Environmental descriptors

We characterized the landscape around the aquatic habitats using the following variables: (i) distance of the aquatic habitat from the nearest forest fragment; (ii) distance of the aquatic habitat from the next nearest water body and (iii) percentage of area around the aquatic habitat that is pasture (Table 1). Variables i and ii were measured in the field when they were less than 200 m, and from satellite images using Google Earth (version 6.1.0.5001) when greater than 200 m. Variable iii was visually estimated for the area encompassed by a circle with a radius of 50 m from the center of each aquatic habitat.

**Table 1:** Variation in environmental descriptors among the sampled aquatic habitats.

	Mean	Standard deviation	Coefficient of variation (%)	Min—Max
**VHE (%)**	28.07	22.60	80.51	0–81
**STA (m**^**2**^**)**	18.60	45.38	243.96	0–247.95
**PPM (%)**	23.68	25.62	108.17	0–90
**PSP (%)**	57.89	31.87	55.05	0–100
**DFF (m)**	163.18	320.08	196.15	0–1420
**DNH (m)**	104.86	211.84	202.02	0–985
**DO (mg/L)**	3.85	0.85	22.21	2.53–5.42
**pH**	7.97	0.95	11.89	6.35–10.48
**CON (mS/cm)**	0.10	0.10	107.97	0–0.38

Abbreviations: VHE—proportion of herbaceous vegetation cover in aquatic habitat (%); STA—size of trampled marshy area (m^2^); PPM—proportion of flat level margin (%); PSP—percentage of surrounding pasture (%); DFF—distance to the nearest forest fragment (m); DNH—distance to the nearest aquatic habitat (m); DO—dissolved oxygen in water (mg/L); pH—hydrogen potential of water; CON—water conductivity (mS/cm).

The physical structure of aquatic habitats was characterized by three variables: (i) size (m^2^) of trampled marshy area (the size of areas with some herbaceous vegetation and cattle hoof prints forming small puddles; S2 Fig); (ii) proportion of herbaceous vegetation cover within the aquatic habitat and (iii) proportion of the margin of the aquatic habitat that is at low levels near the water level (Table 1). We measured width and length of the entire area covered by cattle hoof prints at each aquatic habitat and determined the approximate geometric shape of these areas (rectangle, circle or triangle) in order to estimate area (S1 Fig). These areas may be naturally present in some aquatic habitats throughout the year, or occur once cattle access them or pass through them throughout the year. Whatever the case, the size of these areas may have little variation along the rainy season. The proportion of herbaceous cover was estimated because the variation in the number of calling males could be just a consequence of vegetation, without any association with trampled areas, once trampled areas could also have herbaceous vegetation. Therefore, the percentage of herbaceous vegetation cover in the area of an aquatic habitat was estimated visually, as was the proportion of the margin that was flat level. This latter variable was considered because males of *P*. *mystacalis* are usually found calling from flat level surfaces. These three structural variables were the only variables considered because *P*. *mystacalis* is known to occur in a wide variety of aquatic habitats (*e*.*g*. large and small ponds and marshes [4,35,36]; RMP pers. obs.), but almost always in shallow marshy areas [35,37]. Access of cattle to aquatic habitats was recorded as binary data (1 = cattle present; 0 = cattle absent).

The amount of dissolved oxygen, pH and conductivity of the water (Table 1) were measured, using a Horiba U-10 Multiparameter Meter, because these parameters are known to influence tadpole development and to vary among aquatic habitats, whether cattle had access or not [20,38].

### Data analyses

We used occupancy models to assess whether the presence of cattle and trampled marshy areas could explain the occupancy of aquatic habitats by *Pseudopaludicola mystacalis* [39]. Because amphibian detection probabilities (*P*) in aquatic habitats are usually not constant (*e*.*g*. [40]), we used the number of days from the first sampling (*i*.*e*. the beginning of the rainy season) and the hour when we performed the sampling as variables to account for seasonal and daily variation in detection probabilities, respectively. Hour of sampling was transformed into a decimal for further analysis. As occupancy variables (*ѱ*), we used presence or absence of cattle (1 & 0) and presence or absence of trampled marshy areas (1 & 0). Using these variables we constructed models of all 16 of their possible combinations. Goodness of fit of our global and best models were assessed by Pearson’s chi-square (*X*^2^) using parametric bootstrapping [41] (S3 Table). We selected the model that best predicts occupancy based on Akaike’s information criterion, with correction for small sample sizes (AICc; [42]). We also assessed the relative importance of each variable (*w*+; [42]) in contributing to the detection and occupancy probabilities by summing the Akaike weights (AICc*w*) of all of the models where a specific variable was present [42]. The estimates of each variable were averaged from all of the models accounting for model selection uncertainty [42].

We assessed the environmental variables that best predicted the abundance of *Pseudopaludicola mystacalis* by using N-mixture models for point count data [43]. First, we standardized all environmental variables by subtracting the mean from each value and dividing by the standard deviation [44]. We thus searched for collinearity among environmental variables using the variance inflation factor (VIF; [45]; S4 Table). Again, the goodness of fit of our global and best model were assessed with Pearson’s chi-square using a parametric bootstrapping [41] (S3 Table). We used quasi-likelihood information criteria (QAICc) to select the best models because the global model did not fit any of the available distributions sufficiently (*i*.*e*. Poisson, Negative Binomial and Zero Inflated Poisson; S3 Table). To assess whether variables of the structure of aquatic habitats were better predictors than variables from water quality or landscape, we constructed models with only structural variables, only landscape variables and only water quality variables, plus we constructed models with all possible combinations of these variables and a model without an abundance (λ) variable. We also used all possible combinations of detection variables within those models. This procedure resulted in 32 models that were subjected to model selection based on QAICc values [42]. To specifically assess which variables from the best models were the best predictors of the abundance of calling males, we constructed models with all possible combinations of all variables present in the best predictor models, and assessed their relative importance (*w*+; [42]) by summing the values of their Akaike weights (QAICc*w*) from each model where a given variable was present [42]. We also assessed the average estimates of these variables accounting for model selection uncertainty [42].

All analyses were conducted using the software R 3.1.1 [46]. Standardization of variables and the VIF analysis were performed using the functions *decostand()* and *vif*.*cca()*, respectively, of R package 'vegan' [47]. Model construction was done using the functions *occu()*, for detection/nondetection data, and *pcount()*, for point count data, both from R package 'unmarked' [48]. The model selection procedures were performed using the function *aictab()* from R package 'AICmodavg' [49] and the relative importance of variables and average estimates was assessed using the function *model*.*avg()* from R package 'MuMIn' [50]. The construction of models with all possible combinations of the variables from the best model for predicting the abundance of calling males was done using the function *dredge()* from R package 'AICmodavg' [49]. Goodness of fit tests were performed using the functions *mb*.*gof*.*test()* and *Nmix*.*gof*.*test()* from R package 'AICmodavg' [49].

## Results

### The influence of cattle and their trampled marshy areas on habitat occupancy

The best predictive models (ΔAICc < 2) of occupancy of aquatic habitats by *Pseudopaludicola mystacalis* included all occupancy and detection variables (Table 2). Although the two detection variables were included in the best models, the hour of day was much more important than the day from the beginning of rainy season (Table 3). Sampling hour varied from 4:00 p.m. to 10:50 p.m., and the species had a higher chance of being detected prior to nightfall (*i*.*e*. before 07:00 p.m.; mean 06:48 p.m.; Table 4). Similarly, the species had a better chance of being found calling between the beginning and the middle of the rainy season (mean presence in day 89 of 188 days; Table 4). Both the presence of cattle and trampled marshy areas were important in predicting the occupancy of *P*. *mystacalis*, but the presence of trampled marshy areas was most important (Table 3). The best model predicted the chance of occupancy of an aquatic habitat by *P*. *mystacalis* to be 0.03% (SE = ±0.65) in habitats without trampled marshy areas or cattle access, but 31.34% (SE = ±15.17) in habitats with only cattle access but no trampled marshy areas, and 82,73% (SE = ± 9,38) in habitats with cattle access and trampled marshy areas. In fact, most of the habitats where *P*. *mystacalis* occurred had cattle access and trampled marshy areas (89%, S2 Table). It should be noted that trampled marshy areas only occurred in habitats where cattle had access (S2 Table).

**Table 2:** Model selection data for occupancy by *Pseudopaludicola mystacalis*.

	K	AICc	ΔAICc	AICc*w*
***P*(HOU); ѱ(PCT + PTA)**	5	102.73	0.00	0.31
***P*(HOU + DAY); ѱ(PCT + PTA)**	6	102.90	0.16	0.29
***P*(HOU + DAY); ѱ(PTA)**	5	104.07	1.34	0.16
***P*(DAY); ѱ(PTA)**	4	104.11	1.38	0.16
***P*(HOU); ѱ(PCT)**	4	107.07	4.34	0.04
***P*(HOU + DAY); ѱ(PCT)**	5	107.22	4.48	0.03
***P*(DAY); ѱ(PCT + PTA)**	5	109.96	7.23	0.01

Abbreviations: K—number of parameters; AICc—corrected Akaike’s Information Criteria; ΔAICc—difference in corrected Akaike’s Information Criteria; AICcw—weights of corrected Akaike’s Information Criterion; HOU—hour of the day; DAY—day from the beginning of the rainy season; PCT—presence of cattle; PTA—presence of trampled marshy area. Only models with AICc*w* > 0.00 are shown.

**Table 3:** Relative importance (w+) and average estimates of detection (P) and occupancy variables (ѱ) for occurrence of Pseudopaludicola mystacalis.

	*w+*	Estimate	Standard Error
**Intercept (*P*)**	-	6.591	2.21
**HOU (*P*)**	0.98	-18.035	6.92
**DAY (*P*)**	0.49	-0.004	0.005
**Intercept (ѱ)**	-	-6.244	33.894
**PTA (ѱ)**	0.93	2.447	1.186
**PCT (ѱ)**	0.68	5.308	33.951

Abbreviations: *w*+—sum of the AICc*w* of the models where the parameter was present; HOU—hour of the day; DAY—day from the beginning of the rainy season; PCT—presence of cattle; PTA—presence of trampled marshy area.

**Table 4:** Model selection for abundance of *Pseudopaludicola mystacalis*.

	K	QAICc	ΔQAICc	QAICc*w*
***P*(.); λ(STR + LAN)**	9	126.64	0.00	0.44
***P*(DAY); λ(STR + LAN)**	10	127.40	0.76	0.30
***P*(HOU); λ(STR + LAN)**	10	129.06	2.42	0.13
***P*(HOU + DAY); λ(STR + LAN)**	11	130.08	3.44	0.08
***P*(.); λ(STR)**	6	133.13	6.49	0.02
***P*(DAY); λ(STR)**	7	134.04	7.40	0.01
***P*(.); λ(STR + LAN + WAT)**	12	134.91	8.27	0.01
***P*(HOU); λ(STR)**	7	135.11	8.47	0.01

Abbreviations: K—number of parameters; QAICc—corrected Quasi-Akaike’s Information Criteria; ΔQAICc—difference in corrected Quasi-Akaike’s Information Criteria; QAICcw—weights of corrected Quasi-Akaike’s Information Criteria; HOU—hour of the day; DAY—day from the beginning of the rainy season; STR—variables from physical structure of aquatic habitats, which includes proportion of herbaceous vegetation cover, size of trampled marshy area and proportion of flat level margin; LAN—variables from the surrounding landscape, which includes distance from nearest aquatic habitat, proportion of surrounding pasture and distance from nearest forest fragment; WAT—variables from water quality, which includes dissolved oxygen in water, hydrogen potential of water (pH) and water conductivity. Only models with QAICc*w* > 0.00 are shown. Estimative of *ĉ* for the global model was 3.98.

### Relative importance of aquatic habitat, landscape and water quality variables in determining the abundance of calling *Pseudopaludicola mystacalis*

Neither of the two best models for predicting the abundance of calling males (ΔQAICc < 2) included any of the detection variables, but both included the day from the beginning of the rainy season (Table 4). These models also included predictor variables from both landscape and structure of aquatic habitats. Because models with and without detection variables were both good at predicting the abundance of calling males, we assessed the relative importance of variables using the best model, that is, with no detection variables. We found three variables, two from structure and one from landscape, to be most important (Table 5). These predictors were the size of trampled marshy areas and the proportion of herbaceous vegetation cover within the aquatic habitat and the distance to the nearest aquatic habitat (Table 4).

**Table 5:** Relative importance (w+) and average estimates of detection (P) and abundance variables (λ) from the variables present in the best models (ΔQAICc < 2) for abundance of Pseudopaludicola mystacalis.

	*w+*	Estimate	Standard Error
**Intercept (*P*)**	-	-2.175	0.796
**DAY (*P*)**	0.55	-0.002	0.002
**Intercept (λ)**	-	0.890	0.865
**DNH (λ)**	1	-4.292	1.164
**HEV (λ)**	0.98	0.612	0.143
**STA (λ)**	0.92	0.232	0.087
**PSP (λ)**	0.36	0.169	0.249
**DFF (λ)**	0.25	-0.08	0.174
**PPM (λ)**	0.21	0.026	0.066

Abbreviations: *w*+—sum of the QAICc*w* of the models where the parameter was present; DNH—distance from nearest aquatic habitat; HEV—proportion of herbaceous vegetation cover; STA—size of trampled marshy area; PSP—proportion of surrounding pasture; DFF—distance from nearest forest fragment; PPM—proportion of flat level margin. Estimative of *ĉ* for the model was 3.66.

## Discussion

Our results support the hypothesis that males of *Pseudopaludicola mystacalis* select habitats with marshy areas trampled by cattle. However, the proportion of herbaceous vegetation cover and the proximity of other humid environments were also important. We showed that, in highly deforested regions with mostly livestock activities, the spatial distribution of *P*. *mystacalis* is most influenced by environmental features related to grazing management. More importantly, the presence of cattle, and consequently trampled marshy areas, is a determinant for habitat occupation by *P*. *mystacalis*.

Given that we found the presence of cattle and trampled marshy areas to be important predictors of site occupancy, we suggest that cattle are the main drivers of the occurrence of *Pseudopaludicola mystacalis*. Cattle can create small puddles by trampling marshy areas at the edges of aquatic habitats forming small puddles, which provide suitable habitats for the development of the tadpoles of *P*. *mystacalis* [26]. Furthermore, these puddles can increase the relative humidity of the microhabitat making them more suitable for calling males, which is particularly important for anurans that call during the day in open areas, such as *P*. *mystacalis* [35]. Additionally, the observed positive relationship between the abundance of males of *P*. *mystacalis* and the size of trampled marshy areas may indicate an individual-area relationship [51]. This relationship predicts that as habitat area increases, species abundance should also increase, mostly because of increased physical space and resources. In this case, we believe that larger trampled marshy areas likely provide a greater number of cattle hoof prints (*i*.*e*. small puddles), and thus a greater number of puddles available as calling sites and for tadpole development. In this way cattle may have a direct positive effect on the reproductive success of *P*. *mystacalis*.

Most of the effects of cattle grazing on freshwater diversity are related to the alteration of vegetation by grazing activity [20] or changes in water quality [38] while hoof prints of large animals usually have trivial impacts on other species [52]. However, when these large animals are in high densities, such as the case with cattle, these effects may be greater [52,53]. For example, livestock played an important role in the success of the invasive anuran *Rhinella marina* in Australia [25]. In that case, cattle hoof prints at the edges of ponds provided shelter from desiccation for adults and juveniles of *R*. *marina*, thereby enhancing the survival of these individuals. In the sampled area, cattle are creating microhabitats similar to those used by *P*. *mystacalis* for calling and reproduction in natural habitats. The high occupancy of these new created microhabitats could represent a plastic response to these new environmental conditions. However, despite these positive impact of cattle on site occupancy and abundance of *P*. *mystacalis*, we still do not know what long-term effects such livestock activities can have on populations of this species or on pond communities in general.

Greater proportions of herbaceous vegetation cover in aquatic habitats also favored *P*. *mystacalis*. Although these vegetation types are mostly grasses, they can provide shelter from predators [16,17] and protection from solar radiation, especially for small anurans (about 15mm snout-vent length, [54]) that mostly call before nightfall, such as *P*. *mystacalis* [35]. Additionally, because pastures are essentially grass fields with no canopy cover, a greater proportion of herbaceous vegetation in aquatic habitats located within pastures could be expected. Another possible explanation for this relationship is that greater herbaceous vegetation cover actually reflects the proportion of shallow marshy areas in a particular aquatic habitat (*e*.*g*. depth between 10 and 15 cm), since herbaceous vegetation does not grow in deeper areas (RMP pers. obs).

Despite our predictions that landscape variables would be less important than the structural variables in predicting the abundance of calling males, we found distance to the nearest aquatic habitat to be a very important predictor in our models. This variable has already been considered important for anuran metacommunity structure in the same region [55] and, indeed, *Pseudopaludicola mystacalis* was strongly influenced by this variable in previous work (referred to as *Pseudopaludicola falcipes* 1 in Prado et al [55]). *P*. *mystacalis* is a very small frog only found in open areas with high temperatures and high risk of desiccation. Although other species from this genus are known to be resistant to desiccation [56], pastures could be harsher than natural open areas because constant grazing and trampling may reduce soil vegetation cover, then increasing soil temperature. In such environment, a great availability of aquatic habitats in the landscape should be important to maintain viable populations. Thus, in landscapes with high quality habitats (*i*.*e*. great availability of small puddles in aquatic habitats) and less permeable matrix, it is expected that individuals are less prone to disperse between habitats than in natural landscapes [57]. In this scenario, the proximity between habitats should favors dispersal, explaining the higher abundance found in aquatic habitats with other aquatic habitats nearby.

The occurrence of proximate aquatic habitats is apparently not directly related to grazing management. However, some recent studies have suggested that increasing land use, such as agricultural and livestock activities, is increasing the availability of lentic aquatic habitats [58,59]. Specifically, land owners frequently construct artificial ponds or dams as a water source for livestock (*e*.*g*. [4,5,56]). This could mean that aquatic habitats located within pastures would be more likely to be near other aquatic habitats than habitats located in non-pasture landscapes. Indeed, we observed that the shortest distances to nearest aquatic habitats occurred in habitats with cattle access (S2 Fig). The increased dispersal could increase gene flow and, although it can reduce genetic variability among local populations, it also can decrease rates of homozygosity, increasing fitness of individuals [60]. Additionally, increased dispersal can prevent local extinctions because of the constant arrival of individuals through dispersion [61]. Therefore, aquatic habitats within pasture areas could harbor greater populations of *P*. *mystacalis* and other amphibian species. Nonetheless, this increased dispersal still lacks empirical confirmation.

Finally, our results show a strong contribution by cattle to facilitating the occurrence of *Pseudopaludicola*. *mystacalis*. It has already been shown in Brazil that areas of pasture and sugarcane crops harbor different species and have different species richness than native habitats [62]. These differences are mainly attributed to changes in environmental features such as water quality and hydroperiod [62]. Our study shows that some species can actually be directly influenced by land use. In our case, cattle are directly influencing habitat use by *P*. *mystacalis*. Additionally, two other species of *Pseudopaludicola*, *Pseudopaludicola ternetzi* and *P*. *atragula*, are usually found in syntopy with *P*. *mystacalis* in the study region [4,37], although they are less frequent. These two species also appear to benefit from small puddles created by cattle trampling for reproduction (RMP pers. obs). This leads us to hypothesize that cattle are likely to have an influence on the distribution of these two species as well. However, the effects of cattle grazing in amphibian communities may vary among species [20,63]. Species which are dependent of arboreal and shrubby vegetation for reproduction could be negatively impacted by cattle grazing, while species resistant to desiccation and not dependent of complex vegetacional structures, such as *P*. *mystacalis*, may be favored. We believe that population based studies, such as this one, can help to better plan and refine new and already existing conservation strategies (*e*.*g*. [4,5]) in these highly altered landscapes.

## Supporting Information

S1 Fig Aquatic habitat with marshy area modified by cattle. A: Example of an aquatic habitat with a marshy area modified by cattle trampling. B: Example of the small puddles formed by cattle hoof prints. C and D are the same images but with the trampled marshy area and the small puddles indicated by white dotted lines.
(TIF)

S2 Fig The difference in distance to the nearest aquatic habitat between habitats with and without cattle access. Because there is a possibility that pasture areas have more lentic aquatic habitats than non-pasture habitats, we performed a Kruskal-Wallis test (the data did not meet assumptions of normality and homogeneity of variances even after transformation) to assess whether the distance to the nearest aquatic habitat was greater in habitats without cattle access (*i*.*e*. non-pasture areas). The result was statistically significant (*X*^*2*^ = 8.011, p = 0.004) and supports this hypothesis. However, we still cannot say for sure that the shorter distances to other aquatic habitats in habitats with cattle access is a consequence of higher habitat availability in pastures.
(TIF)

S1 Table Number of pairs of habitats and Pearson’s r in nine distance classes. (DOCX)

S2 Table Geographic coordinates, abundance variables, occupancy variables, detection variables and count data for each sampled aquatic habitat at each sampling occasion. (DOCX)

S3 Table Goodness of fit test for the global and best models of occupancy and point count data for spatial distribution of *Pseudopaludicola mystacalis* in northwestern São Paulo State in southeastern Brazil. (DOCX)

S4 Table Results of the variance inflation factor analyses (VIF). (DOCX)
