# Supplementary material for: Effects of Grazing Management and Cattle on Aquatic Habitat Use by the Anuran Pseudopaludicola mystacalis in Agro-Savannah Landscapes
Source: PLoS One. 2016 Sep 22;11(9):e0163094. doi: 10.1371/journal.pone.0163094 (PMC5033334; doi:10.1371/journal.pone.0163094)
Supplement: S1 Table — (DOCX) [file pone.0163094.s003.docx]

Because studies in real landscapes often lack independence among samples [1], we verified the degree of spatial autocorrelation in our data constructing Mantel Correlograms [2] for our occupancy and abundance data. The Correlograms were built in the software SAM [3]. The degree of spatial autocorrelation was measured by the correlation between Euclidean and geographical distance between all possible pair of habitats within nine distance classes. The distance classes were chosen arbitrarily to represent possible connection by dispersal. Mostly, we were interested in spatial autocorrelation between the closest pairs of habitats (< 1 km). We found no spatial autocorrelation these distance classes.

**S1 Table. Number of pairs of habitats and Pearson’s r in nine distance classes.**

| **Distance Class** | **Number of pairs of habitats** | **Distance Centroid (km)** | **Pearson’s r** | |
| --- | --- | --- | --- | --- |
|  |  |  | **Abundance data** | **Occupancy data** |
| 0 – 0.2 km | 16 | 0.1 | 0.032 | 0.125 |
| 0.2 – 0.5 km | 18 | 0.35 | -0.069 | -0.013 |
| 0.5 – 1 km | 36 | 0.75 | -0.053 | -0.006 |
| 1 – 5 km | 174 | 3 | -0.07 | 0.048 |
| 5 – 10 km | 34 | 7.5 | 0.018 | 0.014 |
| 10 – 20 km | 122 | 15 | -0.059 | -0.062 |
| 20 – 40 km | 204 | 30 | 0.252 | 0.329 |
| 40 – 60 km | 328 | 50 | 0.032 | -0.049 |
| 60 – 80 km | 298 | 70 | -0.034 | -0.148 |

**References.**

1. Peres-Neto PR, Legendre P. Estimating and controlling for spatial structure in the study of ecological communities. Glob Ecol Biogeogr. 2010;19: 174–184. doi:10.1111/j.1466-8238.2009.00506.x

2. Legendre P, Legendre L. Numerical Ecology. 3rd ed. Developments in Environmental Modelling. Oxford: Elsevier; 2012.

3. Rangel TF, Diniz-Filho J a F, Bini LM. SAM: A comprehensive application for Spatial Analysis in Macroecology. Ecography (Cop). 2010;33: 46–50. doi:10.1111/j.1600-0587.2009.06299.x
