# Supplementary material for: Effects of Grazing Management and Cattle on Aquatic Habitat Use by the Anuran Pseudopaludicola mystacalis in Agro-Savannah Landscapes
Source: PLoS One. 2016 Sep 22;11(9):e0163094. doi: 10.1371/journal.pone.0163094 (PMC5033334; doi:10.1371/journal.pone.0163094)
Supplement: S2 Table — (DOCX) [file pone.0163094.s004.docx]

**S2 Table. Geographic coordinates, abundance variables, occupancy variables, detection variables and count data for each sampled aquatic habitat at each sampling occasion.**

| **COD** | **LAT** | **LONG** | **Abundance Variables** | | | | | | | | | **Occupancy Variables** | | **Abundance of *P. mystacalis*** | | | **Detection Variables** | | | | | |
| --- | --- | --- | --- | --- | --- | --- | --- | --- | --- | --- | --- | --- | --- | --- | --- | --- | --- | --- | --- | --- | --- | --- |
|  |  |  |  |  |  |  |  |  |  |  |  |  |  |  |  |  | **HOU** | | | **DAY** | | |
|  |  |  | **VHE** | **DO** | **pH** | **CON** | **PSP** | **DFF** | **DNH** | **STA** | **PPM** | **PCT** | **PTA** | **S1** | **S2** | **S3** | **S1** | **S2** | **S3** | **S1** | **S2** | **S3** |
| **BB1** | 20°54'55,4''S | 49°25'30,2''W | 0.09 | 3.83 | 8.41 | 0.043 | 0 | 6 | 42 | 0 | 0 | 0 | 0 | 0 | 0 | 0 | 05:50:00 | 07:05:00 | 08:00:00 | 15 | 32 | 164 |
| **BB2** | 20°55'32,0''S | 49°25'19,0''W | 0 | 4.47 | 9.36 | 0.124 | 0 | 30 | 86 | 0 | 0 | 0 | 0 | 0 | 0 | 0 | 06:35:00 | 06:13:00 | 08:22:00 | 15 | 32 | 164 |
| **BB3** | 20°55'53,4''S | 49°24'53,2''W | 0.5 | 4.25 | 9.23 | 0.126 | 0 | 4 | 985 | 0 | 0 | 0 | 0 | 0 | 0 | 0 | 06:20:00 | 05:45:00 | 08:48:00 | 15 | 32 | 164 |
| **CAT1** | 21°02'22,8''S | 49°03'51,3''W | 0.21 | 3.16 | 7.73 | 0.055 | 0 | 1290 | 1 | 0 | 10 | 0 | 0 | 0 | 0 | 0 | 08:05:00 | 06:34:00 | 07:45:00 | 52 | 130 | 187 |
| **CAT2** | 21°03'45,2''S | 49°04'36,9''W | 0.3 | 2.93 | 9.87 | 0.124 | 0 | 175 | 570 | 0 | 0 | 0 | 0 | 0 | 0 | 0 | 07:20:00 | 07:20:00 | 06:00:00 | 52 | 130 | 187 |
| **CAT3** | 21°03'56,3''S | 49°04'20,2''W | 0.1 | 3.02 | 7.63 | 0.007 | 0 | 7 | 320 | 0 | 0 | 0 | 0 | 0 | 0 | 0 | 07:00:00 | 07:34:00 | 07:10:00 | 52 | 130 | 187 |
| **CAT4** | 21°04'08,8''S | 49°04'20,8''W | 0.81 | 3.65 | 6.35 | 0.007 | 55 | 1 | 0 | 1.82 | 0 | 1 | 1 | 11 | 0 | 0 | 05:47:00 | 08:27:00 | 06:46:00 | 88 | 130 | 187 |
| **CAT5** | 21°04'12''S | 49°04'14,8''W | 0.49 | 3.51 | 8.14 | 0.087 | 65 | 23 | 0 | 26.80 | 30 | 1 | 1 | 7 | 0 | 0 | 06:22:00 | 07:55:00 | 06:23:00 | 88 | 130 | 187 |
| **ICE1** | 20°21'50,3''S | 49°14'18,9''W | 0.49 | 3.16 | 8.14 | 0.28 | 50 | 46 | 18 | 1.25 | 10 | 1 | 1 | 1 | 9 | 0 | 05:40:00 | 07:25:00 | 10:50:00 | 28 | 86 | 154 |
| **ICE10** | 20°21'45,8''S | 49°14'13,7''W | 0.09 | 2.83 | 7.14 | 0.017 | 90 | 32 | 0 | 4.9 | 90 | 1 | 1 | 0 | 2 | 1 | 07:33:00 | 04:00:00 | 07:12:00 | 30 | 125 | 176 |
| **ICE11** | 20°21'46,6''S | 49°14'10,9''W | 0.21 | 3.71 | 9.39 | 0.061 | 100 | 68 | 110 | 7 | 30 | 1 | 1 | 0 | 1 | 0 | 06:35:00 | 04:10:00 | 10:15:00 | 30 | 125 | 176 |
| **ICE12** | 20°21'18,8''S | 49°12'17,0''W | 0.06 | 4.74 | 7.35 | 0.035 | 100 | 30 | 1 | 5.25 | 30 | 1 | 1 | 0 | 2 | 0 | 06:10:00 | 04:30:00 | 09:50:00 | 30 | 125 | 176 |
| **ICE13** | 20°21'22,6''S | 49°12'07,5''W | 0.49 | 3.14 | 7.84 | 0 | 80 | 0 | 0 | 2.7 | 0 | 1 | 1 | 5 | 2 | 0 | 08:10:00 | 07:04:00 | 07:48:00 | 28 | 125 | 176 |
| **ICE2** | 20°21'26,1''S | 49°11'57,7''W | 0.27 | 2.54 | 8.98 | 0.104 | 50 | 0 | 0 | 48.51 | 10 | 1 | 1 | 1 | 6 | 0 | 07:00:00 | 05:21:00 | 10:25:00 | 28 | 86 | 154 |
| **ICE3** | 20°21'51,9''S | 49°11'26,4''W | 0.54 | 2.83 | 7.25 | 0.191 | 70 | 18 | 22 | 91.76 | 10 | 1 | 1 | 1 | 2 | 0 | 07:25:00 | 05:55:00 | 10:05:00 | 28 | 86 | 154 |
| **ICE4** | 20°21'47,8''S | 49°11'36,1''W | 0.27 | 3.22 | 7.69 | 0.02 | 100 | 350 | 1.7 | 0 | 50 | 1 | 0 | 1 | 6 | 0 | 07:05:00 | 06:58:00 | 09:03:00 | 7 | 69 | 154 |
| **ICE5** | 20°22'07,8''S | 49°11'33,9''W | 0.5 | 3.49 | 7.66 | 0.054 | 100 | 330 | 270 | 1.92 | 50 | 1 | 1 | 0 | 0 | 0 | 07:45:00 | 07:33:00 | 06:30:00 | 7 | 69 | 154 |
| **ICE6** | 20°22'08,1''S | 49°11'33,3''W | 0.63 | 2.99 | 8.56 | 0.006 | 50 | 170 | 0 | 247.95 | 30 | 1 | 1 | 27 | 5 | 16 | 08:55:00 | 06:10:00 | 07:02:00 | 7 | 69 | 154 |
| **ICE7** | 20°22'02,2''S | 49°12'04,0''W | 0.5 | 3.84 | 7.83 | 0.016 | 60 | 5 | 0 | 12 | 0 | 1 | 1 | 5 | 11 | 6 | 09:00:00 | 06:08:00 | 08:58:00 | 30 | 125 | 176 |
| **ICE8** | 20°22'03,9''S | 49°12'08,9''W | 0.42 | 3.57 | 7.7 | 0.023 | 90 | 150 | 0 | 9.45 | 30 | 1 | 1 | 10 | 5 | 0 | 08:30:00 | 06:37:00 | 08:23:00 | 30 | 125 | 176 |
| **ICE9** | 20°21'53,1''S | 49°11'37,5''W | 0.28 | 3.19 | 6.87 | 0.026 | 70 | 3 | 10 | 8.25 | 50 | 1 | 1 | 0 | 7 | 6 | 07:10:00 | 07:40:00 | 07:50:00 | 30 | 125 | 176 |
| **NG1** | 20°25'23,5''S | 49°16'44,5''W | 0.63 | 4.62 | 7.44 | 0.021 | 50 | 45 | 6 | 25.35 | 70 | 1 | 1 | 6 | 1 | 0 | 06:25:00 | 08:10:00 | 07:14:00 | 47 | 127 | 185 |

**S2 Table. Continued.**

| **COD** | **LAT** | **LONG** | **Abundance Variables** | | | | | | | | | **Occupancy Variables** | | **Abundance of *P. mystacalis*** | | | **Detection Variables** | | | | | |
| --- | --- | --- | --- | --- | --- | --- | --- | --- | --- | --- | --- | --- | --- | --- | --- | --- | --- | --- | --- | --- | --- | --- |
|  |  |  |  |  |  |  |  |  |  |  |  |  |  |  |  |  | **HOU** | | | **DAY** | | |
|  |  |  | **VHE** | **DO** | **pH** | **CON** | **PSP** | **DFF** | **DNH** | **STA** | **PPM** | **PCT** | **PTA** | **S1** | **S2** | **S3** | **S1** | **S2** | **S3** | **S1** | **S2** | **S3** |
| **NG2** | 20°26'22,1''S | 49°16'38,4''W | 0.05 | 4.71 | 7.53 | 0.044 | 60 | 5 | 15 | 0 | 0 | 1 | 0 | 0 | 0 | 0 | 07:03:00 | 07:17:00 | 07:50:00 | 47 | 127 | 185 |
| **NG3** | 20°26'53,95S | 49°15'27,2''W | 0.14 | 4.69 | 8.43 | 0.05 | 40 | 60 | 8 | 0 | 0 | 1 | 0 | 1 | 5 | 0 | 07:53:00 | 05:45:00 | 08:35:00 | 47 | 127 | 185 |
| **NG4** | 20°26'42,1''S | 49°16'03,9''W | 0 | 5.17 | 7.74 | 0.022 | 30 | 0 | 160 | 0 | 70 | 0 | 0 | 0 | 0 | 0 | 08:45:00 | 06:19:00 | 09:00:00 | 47 | 127 | 185 |
| **NG5** | 20°26'37,3''S | 49°15'14,6''W | 0 | 4.37 | 10.48 | 0.079 | 90 | 310 | 311 | 0 | 0 | 1 | 0 | 0 | 0 | 0 | 09:00:00 | 05:43:00 | 09:25:00 | 47 | 127 | 185 |
| **NI1** | 21°04'52,6''S | 49°31'09,1''W | 0.56 | 3.55 | 7.49 | 0.016 | 60 | 10 | 20 | 76 | 50 | 1 | 1 | 2 | 8 | 8 | 06:30:00 | 06:50:00 | 07:50:00 | 1 | 54 | 147 |
| **NI2** | 21°04'50,9''S | 49°31'11,5''W | 0.45 | 3.18 | 6.79 | 0.191 | 80 | 40 | 90 | 6.9 | 50 | 1 | 1 | 0 | 1 | 0 | 07:35:00 | 03:37:00 | 08:40:00 | 1 | 101 | 147 |
| **NI3** | 21°04'40,0''S | 49°32'22,8''W | 0.45 | 3.87 | 6.75 | 0.307 | 80 | 30 | 45 | 0 | 10 | 1 | 0 | 0 | 0 | 0 | 08:43:00 | 08:10:00 | 06:15:00 | 1 | 54 | 147 |
| **NI4** | 21°04'43,8''S | 49°32'20,2''W | 0.4 | 4.34 | 7.44 | 0.098 | 70 | 30 | 4 | 0 | 0 | 1 | 0 | 0 | 0 | 0 | 09:15:00 | 08:35:00 | 06:43:00 | 1 | 54 | 147 |
| **POT1** | 21°06'27,6''S | 49°20'13''W | 0.36 | 2.53 | 7.35 | 0.169 | 45 | 2 | 4 | 88.8 | 10 | 1 | 1 | 0 | 0 | 0 | 06:40:00 | 09:22:00 | 07:55:00 | 77 | 135 | 188 |
| **POT2** | 21°07'08,1''S | 49°20'23,5''W | 0.09 | 2.75 | 7.04 | 0.06 | 65 | 170 | 0 | 0.18 | 70 | 1 | 1 | 0 | 0 | 0 | 04:10:00 | 09:02:00 | 08:25:00 | 77 | 135 | 188 |
| **RP1** | 20°46'49,7''S | 49°18'50,1''W | 0 | 4.58 | 8.2 | 0.384 | 90 | 191 | 661 | 0 | 30 | 1 | 0 | 0 | 0 | 0 | 05:40:00 | 08:20:00 | 05:50:00 | 22 | 126 | 184 |
| **RP2** | 20°46'18,2''S | 49°18'36,8''W | 0 | 5.3 | 6.68 | 0.37 | 45 | 50 | 40 | 0 | 0 | 1 | 0 | 0 | 0 | 0 | 06:07:00 | 07:55:00 | 06:18:00 | 22 | 126 | 184 |
| **RP3** | 20°46'15,0''S | 49°18'34,7''W | 0.09 | 4.89 | 8.52 | 0.04 | 60 | 200 | 4 | 40.04 | 30 | 1 | 1 | 0 | 0 | 0 | 06:25:00 | 07:32:00 | 06:40:00 | 22 | 126 | 184 |
| **RP4** | 20°45'20,9''S | 49°18'06,1''W | 0.1 | 4.89 | 9.28 | 0.08 | 30 | 750 | 100 | 0 | 0 | 0 | 0 | 0 | 0 | 0 | 09:15:00 | 05:35:00 | 07:14:00 | 22 | 126 | 184 |
| **RP5** | 20°45'06,2''S | 49°17'53,8''W | 0.1 | 5.22 | 7.56 | 0.3 | 90 | 150 | 60 | 0 | 30 | 1 | 0 | 3 | 3 | 1 | 07:50:00 | 06:10:00 | 07:43:00 | 22 | 126 | 184 |
| **RP6** | 20°45'37,7''S | 49°18'20''W | 0 | 5.42 | 8.97 | 0.051 | 85 | 1420 | 20 | 0 | 50 | 1 | 0 | 0 | 0 | 0 | 07:00:00 | 06:40:00 | 08:10:00 | 22 | 54 | 184 |

COD – Aquatic habitat code; LAT – Latitude coordinate of aquatic habitat; LONG – Longitude coordinate of aquatic habitat; VHE – Proportion of herbaceous vegetation cover in aquatic habitat (%); DO – Dissolved oxygen in water (mg/L); pH – Potential Hydrogen; CON – Water conductivity (mS/cm); PSP – Percentage of surrounding pasture (%); DFF – Distance to the nearest forest fragment (m); DNH – Distance to the nearest aquatic habitat (m); STA – Size of trampled marshy area (m²); PPM – Proportion of plan margin (%); PCT – Cattle presence; PTA; presence of trampled marshy area; HOU – hour of the day when we sampled the aquatic habitat; DAY – Number of the day from the first day of sampling; S1, S2, S3 – Sampling occasions.
