# Supplementary material for: Effects of Grazing Management and Cattle on Aquatic Habitat Use by the Anuran Pseudopaludicola mystacalis in Agro-Savannah Landscapes
Source: PLoS One. 2016 Sep 22;11(9):e0163094. doi: 10.1371/journal.pone.0163094 (PMC5033334; doi:10.1371/journal.pone.0163094)
Supplement: S3 Table — (DOCX) [file pone.0163094.s005.docx]

We accessed the fit of our global models by goodness of fit tests based on Pearson’s chi-square (*X²*) using a parametric bootstrap approach with 1000 simulations to obtain P-values. We also accessed an estimate of the overdispersion parameter (*ĉ*) for the model by dividing the observed chi-square statistic by the mean of the statistics obtained from the simulations [1]. The results of occupancy data had little overdispersion (*ĉ* ≈ *1*), thus there was no need to use quasi-likelihood information criteria (QAICc) to further analysis [2]. However, our point count data presented overdispersion (*ĉ >>* 1) in Poisson distributions and underdisperstion (*ĉ <<* 1) in Negative Binomial and Zero Inflated Poisson distribution [2]. Therefore, we used the estimated values of *ĉ* to compute quasi-likelihood information criteria (QAICc) and select the best model. We also performed a goodness of fit tests to the best model to use the new estimated *ĉ* values (in case of overdispersion) to access the relative importance of each variable from the best models [2].

**S3 Table. Goodness of fit test for the global and best models of occupancy and point count data for spatial distribution of *Pseudopaludicola mystacalis* in northwestern São Paulo State in southeastern Brazil.**

| **Type of data** | **Model** | **Distribution** | ***X²*** | **p** | ***ĉ*** |
| --- | --- | --- | --- | --- | --- |
| Occupancy data | Global Model | - | 5.877 | 0.265 | 1.27 |
|  | Best Model | - | 4.421 | 0.355 | 1.1 |
| Point Count data | Global Model | Poisson | 338.019 | 0.002 | 3.98 |
|  |  | Negative Binomial | 4924.886 | 0.389 | 0 |
|  |  | Zero Inflated Poisson | 502.811 | 0.08 | 0 |
|  | Best Model | Poisson | 326.143 | 0.001 | 3.7 |

**References.**

1. MacKenzie DI, Bailey LL. Assessing the fit of site-occupancy models. J Agric Biol Environ Stat. 2004;9: 300–318. doi:10.1198/108571104X3361

2. Burnham KKP, Anderson DRD. Model Selection and Multimodel Inference: A Practical Information-Theoretic Approach (2nd ed) [Internet]. Ecological Modelling. 2002. doi:10.1016/j.ecolmodel.2003.11.004
