# Supplementary material for: Effects of Grazing Management and Cattle on Aquatic Habitat Use by the Anuran Pseudopaludicola mystacalis in Agro-Savannah Landscapes
Source: PLoS One. 2016 Sep 22;11(9):e0163094. doi: 10.1371/journal.pone.0163094 (PMC5033334; doi:10.1371/journal.pone.0163094)
Supplement: S4 Table — (DOCX) [file pone.0163094.s006.docx]

**S4 Table. Results of the variance inflation factor analyses (VIF).**

|  | **VHE** | **DO** | **pH** | **CON** | **PSP** | **DFF** | **DNH** | **STA** | **PPM** |
| --- | --- | --- | --- | --- | --- | --- | --- | --- | --- |
| **VIF value** | 1.763 | 1.386 | 2.017 | 1.384 | 1.513 | 1.220 | 1.732 | 1.452 | 1.692 |

Because all abundance variables had VIF values < 3, we did not exclude any variable from further analysis. VHE – Proportion of herbaceous vegetation cover in aquatic habitat; DO – Dissolved oxygen in water; pH – Potential Hydrogen; CON – Water conductivity; PSP – Percentage of surrounding pasture; DFF – Distance to the nearest forest fragment; DNH – Distance to the nearest aquatic habitat; STA – Size of trampled marshy area; PPM – Proportion of plan margin.
